# Supplementary figures and images for: Adherence barriers and interventions to improve ART adherence in Sub-Saharan African countries: A systematic review protocol
Source: PLoS One. 2022 Jun 15;17(6):e0269252. doi: 10.1371/journal.pone.0269252 (PMC9200354; doi:10.1371/journal.pone.0269252)

**Supplementary file 2. PRISMA 2009 Flow Diagram**


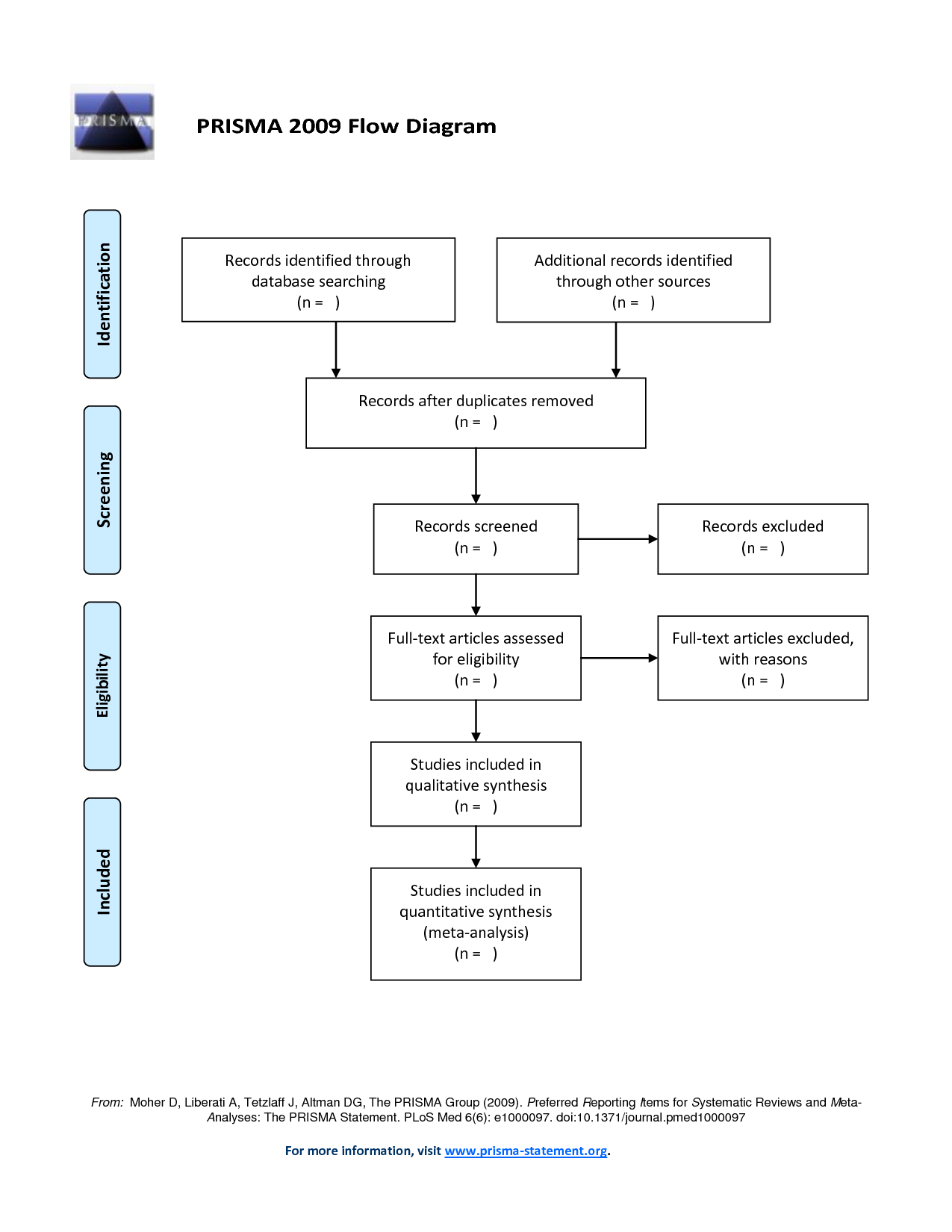

Supplement: S2 File — (DOCX) [file pone.0269252.s002.docx]
