# Supplementary material for: Adherence barriers and interventions to improve ART adherence in Sub-Saharan African countries: A systematic review protocol
Source: PLoS One. 2022 Jun 15;17(6):e0269252. doi: 10.1371/journal.pone.0269252 (PMC9200354; doi:10.1371/journal.pone.0269252)
Supplement: S3 File — (DOCX) [file pone.0269252.s003.docx]

**Supplementary file 3: Medline (Ovid) Search Strategies and Results**

1 test.ti. 173184

2 exp HIV/ 102314

3 exp Acquired Immunodeficiency Syndrome/ 76887

4 exp AIDS-related opportunistic infections/ 21220

5 exp HIV infections/ 294267

6 human immunodeficiency virus*.ti,ab,kf. 91838

7 acquired immunodeficiency syndrome.ti,ab,kf. 20079

8 AIDS.ti,ab,kf. 159137

9 HIV.ti,ab,kf. 329040

10 2 or 3 or 4 or 5 or 6 or 7 or 8 or 9 465353

11 exp Antiviral agents/ 373305

12 exp Anti-HIV agents/ 71150

13 exp Antiretroviral Therapy, Highly Active/ 21988

14 exp HIV integrase inhibitors/ 2740

15 exp HIV protease inhibitors/ 13910

16 exp Reverse Transcriptase Inhibitors/ 33398

17 reverse transcriptase inhibit*.ti,ab,kf. 8626

18 antiviral*.ti,ab,kf. 101021

19 anti-HIV.ti,ab,kf. 13222

20 (antiretroviral* or anti-retro*).ti,ab,kf. 69486

21 exp Nevirapine/ 2795

22 exp Delavirdine/ 196

23 exp Zidovudine/ 9564

24 exp Lamivudine/ 6603

25 (Fosamprenavir* or Atazanavir* or Indinavir* or Nelfinavir* or Saquinavir* or Ritonavir* or Amprenavir* or Darunavir* or Lopinavir* or ritonavir* or kaletra* or Tripanavir* or tipranavir* or nevirapine* or Abacavir* or Stavudine* or Didanosine* or Lamivudine* or Zidovudine* or Zalcitabine* or Combivir* or Trizivir* or tenofovir* or efavirenz* or delavirdine*).ti,ab,kf. 35684

26 HAART.ti,ab,kf. 12298

27 cART.mp. 8331

28 ARV.mp. 3522

29 ART.mp. 126922

30 anti-HIV agents/ or Antiviral Agents/ 132191

31 Anti-Retroviral Agents/ or Antiretroviral Therapy, Highly Active/ 31883

32 11 or 12 or 13 or 14 or 15 or 16 or 17 or 18 or 19 or 20 or 21 or 22 or 23 or 24 or 25 or 26 or 27 or 28 or 29 or 30 or 31 577616

33 10 and 32 137384

34 exp Africa South of the Sahara/ 225419

35 cent* Africa*.ti,ab,kf. 4983

36 east* Africa*.ti,ab,kf. 11306

37 south* Africa*.ti,ab,kf. 51791

38 west* Africa*.ti,ab,kf. 16611

39 34 or 35 or 36 or 37 or 38 254704

40 33 and 39 14224

41 "Treatment Adherence and Compliance"/ 762

42 Medication Adherence/ 21370

43 adher*.ti,ab,kf. 211179

44 retention*.ti,ab,kf. 190213

45 retain*.ti,ab,kf. 220493

46 complian*.ti,ab,kf. 141300

47 comply.ti,ab,kf. 11870

48 complied.ti,ab,kf. 4969

49 noncomplian*.ti,ab,kf. 8739

50 non-complian*.ti,ab,kf. 7185

51 non-adher*.ti,ab,kf. 9925

52 nonadher*.ti,ab,kf. 10417

53 loss to follow-up.ti,ab,kf. 5019

54 lost to follow-up.ti,ab,kf. 19685

55 attrition.ti,ab,kf. 14116

56 41 or 42 or 43 or 44 or 45 or 46 or 47 or 48 or 49 or 50 or 51 or 52 or 53 or 54 or 55 781016

57 40 and 56 3890

58 (Angola* or Benin* or Botswana* or Burkina Faso* or Burundi* or Cameroon* or Cape Verde* or Central African Republic* or Chad* or Comoros* or Congo* or Cote d'Ivoire* or Djibouti* or Equatorial Guinea* or Eritrea* or Ethiopia* or Gabon* or Gambia* or Ghana* or Guinea* or Guinea-Bissau* or Kenya* or Lesotho* or Liberia* or Madagascar* or Malawi* or Mali* or Mauritania* or Mauritius* or Mozambique* or Namibia* or Niger* or Nigeria* or Reunion* or Rwanda* or "Sao Tome adj2 Principe*" or Senegal* or Seychelles* or Sierra Leone* or Somalia* or South Africa* or Sudan* or Swaziland* or Tanzania* or Togo* or Uganda* or Western Sahara* or Zambia* or Zimbabwe*).ti,ab,kf. 1016228

59 39 or 58 1078865

60 33 and 59 18347

61 56 and 60 4589

62 ("26927422" or "27042024" or "23573232" or "25477050" or "30691425" or "31852741" or "34134738" or "33222633" or "33125587").ui. 9

63 61 and 62 7

64 62 not 63 2

65 limit 61 to yr="2010 -Current" 4113
