# Supplementary material for: Adherence barriers and interventions to improve ART adherence in Sub-Saharan African countries: A systematic review protocol
Source: PLoS One. 2022 Jun 15;17(6):e0269252. doi: 10.1371/journal.pone.0269252 (PMC9200354; doi:10.1371/journal.pone.0269252)
Supplement: S5 File — (DOCX) [file pone.0269252.s005.docx]

**Supplementary file 5. Data Extraction Form for Quantitative Research**

**Study Characteristics**

**1.       Author:**

**2.       Year of publication:**

**3.       Journal:**

**4.       Record number:**

**Study Method**

**1.       Study Design:**

| RCT | Quasi-RCT | Longitudinal | Retrospective | Observational | Other |
| --- | --- | --- | --- | --- | --- |
|  |  |  |  |  |  |

**2.       Purpose:**

**3.       Research Questions:**

**Participant Characteristics**

| Country where the study took place | Setting | Population | Sex | Age | Sample size | Ethnicity (if applicable) | socioeconomic status (if applicable) | education level (if applicable) |
| --- | --- | --- | --- | --- | --- | --- | --- | --- |
|  |  |  |  |  |  |  |  |  |

**Pressure Ulcer Prevention Strategies**

**Strategy 1:**

**Strategy 2:**

**Strategy 3:**

**Outcome Measures**

| Outcome Description | Scale/measure |
| --- | --- |
|  |  |
|  |  |
|  |  |

**Study results**

**Dichotomous data**

| Outcome | Intervention ( ) number / total number | Intervention ( )number / total number |
| --- | --- | --- |
|  |  |  |
|  |  |  |
|  |  |  |
|  |  |  |
|  |  |  |

**Continuous data**

| Outcome | Intervention ( ) mean & SD (number) | Intervention (  ) mean & SD (number) |
| --- | --- | --- |
|  |  |  |
|  |  |  |
|  |  |  |

**Authors’ conclusions:**

**Comments:**
