# Supplementary material for: Adherence barriers and interventions to improve ART adherence in Sub-Saharan African countries: A systematic review protocol
Source: PLoS One. 2022 Jun 15;17(6):e0269252. doi: 10.1371/journal.pone.0269252 (PMC9200354; doi:10.1371/journal.pone.0269252)
Supplement: Attachment — Submitted filename: Peer-review to PLoS ONE.docx [file pone.0269252.s006.docx]

Title: Adherence barriers and interventions to improve ART adherence in Sub-Saharan African countries: a systematic review protocol

Dear Editor,

The protocol is generally well drafted and will be valuable input to policy makers working on the area of HIV/AIDS and/or ART in the sub-saharan African countries. Providing concrete and cumulative evidence on barriers to and strategies for ART adherence will also be highly recommended to maximize treatment outcome in the region.

Having said this, I do have few concerns which require due attention while conducting this systematic review and meta-analysis

- Grammatical, syntax and technical problems

**Abstract**

- E.g. Background: Despite efforts to prevent new infections and deaths through antiretroviral therapy (ART)…… Can we prevent new infections with ART? Please rephrase the statement.
- Method: The review will include experimental and quasi-experimental studies such as…….. Please make it concise
- Why do you focus on articles published after 2010?
- The role of case studies and case-control studies for this protocol (interventional approach) should be clarified. It will be better if you include studies with interventional or observational nature (cohort or cross-sectional) ……
- Discussion: this review will provide information **to** policy makers….. better ways of not only retaining patients on treatment but **also** having them adhere to their treatment……

**Method:**

Search strategy requires refinement

- The keywords should be concise and explicit. It is not recommended to use phrases in searching unless it is a medical term or compulsory otherwise.
- Use Medical Subject Headings (MesH) during searching in major databases such as MEDLINE, PubMed. E.g. instead of using sub-saharan African Countries, apply “African, south of the Sahara” to address all countries in the region at once with MeSH.
- You can use truncation (*) to expand the search results as well. E.g. instead of using adulthood, apply adult*
- Make sure that you have prepared a ‘PICO’ tree for effectively addressing all studies conducted in the region.
- Google scholar and CDC are not databases, they are either search engines or directories…..

**Discussion:**

- The discussion is actually shallow. You can explain the rationale of conducting this protocol supported with citations.
- This protocol is an original research protocol??????? It is a systematic review protocol
